# Brabykinin B1 Receptor Antagonism Is Beneficial in Renal Ischemia-Reperfusion Injury

**DOI:** 10.1371/journal.pone.0003050

**Published:** 2008-08-25

**Authors:** Pamella H. M. Wang, Gabriela Campanholle, Marcos A. Cenedeze, Carla Q. Feitoza, Giselle M. Gonçalves, Richardt G. Landgraf, Sonia Jancar, João B. Pesquero, Alvaro Pacheco-Silva, Niels O. S. Câmara

**Affiliations:** 1 Laboratório de Imunologia Clínica e Experimental, Division of Nephrology, Universidade Federal de São Paulo, São Paulo, Brazil; 2 Laboratório de Imunobiologia de Transplantes, Department of Immunology, Universidade de São Paulo, São Paulo, Brazil; 3 Laboratório de Imunofarmacologia, Department of Immunology, Universidade de São Paulo, São Paulo, Brazil; 4 Department of Biophysics, Universidade Federal de São Paulo, São Paulo, Brazil; Instituto Oswaldo Cruz and FIOCRUZ, Brazil

## Abstract

Previously we have demonstrated that bradykinin B1 receptor deficient mice (B1KO) were protected against renal ischemia and reperfusion injury (IRI). Here, we aimed to analyze the effect of B1 antagonism on renal IRI and to study whether B1R knockout or antagonism could modulate the renal expression of pro and anti-inflammatory molecules. To this end, mice were subjected to 45 minutes ischemia and reperfused at 4, 24, 48 and 120 hours. Wild-type mice were treated intra-peritoneally with antagonists of either B1 (R-954, 200 µg/kg) or B2 receptor (HOE140, 200 µg/kg) 30 minutes prior to ischemia. Blood samples were collected to ascertain serum creatinine level, and kidneys were harvested for gene transcript analyses by real-time PCR. Herein, B1R antagonism (R-954) was able to decrease serum creatinine levels, whereas B2R antagonism had no effect. The protection seen under B1R deletion or antagonism was associated with an increased expression of GATA-3, IL-4 and IL-10 and a decreased T-bet and IL-1β transcription. Moreover, treatment with R-954 resulted in lower MCP-1, and higher HO-1 expression. Our results demonstrated that bradykinin B1R antagonism is beneficial in renal IRI.

## Introduction

Renal ischemia and reperfusion injury (IRI) is a leading cause of acute renal failure in both allografts and native kidneys [1]. Inflammation plays an important role in the pathogenesis of renal IRI, through leukocyte activation and expression of adhesion molecules and cytokines [2]–[8]. Therefore, new therapeutic strategies aiming to reduce this inflammatory response could be beneficial.

Bradykinin receptor activation may affect this inflammatory response. Physiological effects of bradykinin are mediated by two transmembrane G-coupled proteins, namely B1 and B2 receptors (B1R and B2R, respectively) [9]. B2R is constitutively expressed under physiological conditions and is responsible for most kinin effects [9]. By contrast, B1R is normally weakly expressed, being strongly up-regulated in the presence of inflammatory stimuli [10], [11] or its natural agonist des-Arg^10^-Kallidin [12]. Its role after activation remains unclear. Several studies have shown that B1R can influence immune responses by modulating T lymphocytes [13], [14] and leukocyte migration [14], [15] and prostaglandins [16], mast cell mediators [17], cytokines [18], [19] and chemokines production [20]–[22].

Regarding renal IRI, we have previously demonstrated that B1R knockout mice were protected against IRI. In the other hand, B2R antagonism [23] or simultaneous receptor knockouts [24] was shown to be deleterious.

This study aimed to analyze the impact of bradykinin B1 and B2R antagonism on renal IRI and to determine the influence of these tratments on renal pro and anti-inflammatory molecules expression.

## Methods

### Animals

Isogenic male B1R, B2R and B1B2R-deficient C57BL/6 mice (B1KO, B2KO and B1B2KO, respectively) aged 8–12 wks (25–28 g) were used. All mice were kindly donated by Prof. João Bosco Pesquero of the Biophysics Department of the Federal University of São Paulo (UNIFESP), Brazil. All animals were housed in individual and standard cages and had free access to water and food. Wild type C57BL/6 mice (B1B2WT), matched for age and sex, were used as control animals. All procedures were previously reviewed and approved by the internal Ethics Committee of the Institution.

### Experimental Model of Renal IRI

Surgery was performed as previously described [3]. Mice were briefly anesthetized with Ketamine-Xylazine (Agribrands do Brazil, São Paulo, Brazil). A midline incision was made and both renal pedicles were cross-clamped for 45 minutes. During the procedure, animals were kept well hydrated with saline and at a constant temperature (∼37°C) by means of a heating pad device. Subsequently, microsurgery clamps were removed, the abdomen closed and animals placed in single cages, warmed by indirect light until fully recovered from anesthesia. Animals were kept under controlled conditions until sacrifice, according to experimental protocol at 4, 24, 48 and 120 hours after renal reperfusion. Fifteen animals (n = 15) were sacrificed at each time point. To serve as controls, ten *sham* animals (n = 10) from each group were subjected to the surgical procedure Groups were broken down for technical and scientific purposes to allow 15 surgeries per day, 5 from each group, namely, 15 IRI surgeries or 15 sham-operated animals per day.

### Analysis of Renal Function

Serum creatinine was used for renal function assessment. Blood samples were collected at 4, 24, 48 and 120 hours post reperfusion from the abdominal inferior cava vein immediately before induced death. Serum samples were analyzed on a Cobas Mira Plus (Roche, Mannhein, Germany), using the modified Jaffé technique.

### Caspase-3 Activity Assay

Kidneys were collected at 24 hours and homogenized in 1 mL of ice-cold 40 mmol/L Tris-HCl pH 7.6 with 1% triton X-100. Extracted kidneys were centrifuged and supernatants collected. Protein extracts (50 µg) were diluted in 1 mL of protease assay buffer (20 mmol/L Pipes, 100 mmol/L NaCl, 10 mmol/L DTT, 1 mmol/L EDTA, 0.1% CHAPS, 10% sucrose, pH 7.2) containing 10 µL of Ac-DEVD-AFC substrate (PharMingen, BD Biosciences, San Jose, USA) and incubated for 1h at 37°C. Samples were analyzed using a fluorimeter (F-2000, Hitachi, Japan) at 505 nm.

### Antagonist and agonist treatments

All treatments were administrated i.p. 30 minutes prior to ischemia. For B1R and B2R antagonism, wild-type animals were treated with R-954 or HOE-140 (200 µg/kg), while for B1R agonism, animals were treated with des-Arg^9^-BK (DABK) at a concentration of 600 µg/kg. Agonist and antagonists were kindly donated by Dr. Pierre Sirois from the Université de Sherbrooke, Québec, Canada. All doses were based on two previously published studies that had used these same compounds for *in vivo* treatment [25], [26].

### Gene Profiles

Kidney samples were quickly frozen in liquid nitrogen. Total RNA was isolated by TRIzol Reagent (Invitrogen, California, USA) methodology. First-strand cDNAs were synthesized using MML-V reverse transcriptase (Promega, Madison, USA). Real-time PCR was performed using TaqMan PCR assays as followed: IL-1β (Mm00434228_m1), IL-4 (Mm00445259_m1), IL-10 (Mm00439616_m1), Bcl-2 (Mm00477631_m1) and Bad (Mm00432042_m1) plus the housekeeper gene hypoxanthine guanine phosphoribosyltransferase (HPRT) (Mm00446968_m1)(Applied Biosystem, California, USA). Real-time PCR was performed for GATA-3, T-bet, MCP-1, HO-1, B1R and B2R expression using SYBR Green assay (Applied Biosystem). In this case, another specific SYBR Green HPRT was used. Sequences of oligonucleotides are depicted in Table 1. Cycling conditions were: 10 minutes at 95°C followed by 45 cycles of 30 seconds at 95°C, 30 seconds at 58°C and 30 seconds at 72°C. Relative quantification of mRNA levels was performed using the comparative threshold cycle method (described in detail in User Bulletin 2; PerkinElmer, Applied Biosystems, Branchburg, NJ, 1997). Briefly, the target gene amount was normalized to the endogenous reference (HPRT) and then compared against a calibrator (sample with the lowest expression, namely, *sham*-operated animals), using the formula 2^−DDCT^. Hence, all data were expressed as an n-fold difference in relation to the expression of matched controls (*sham*). Analyses were performed with the Sequence Detection Software 1.9 (SDS).

**Table 1:** Oligonucleotides sequence.

GENE	FORWARD	REVERSE
GATA-3	5′-GCC TGT GCA AAA GAG ATT TCA GAT-3′	5′-TGA TTC ACA GAG CAT GTA GGC C-3′
T-bet	5′-CCA GTA TCC TGT TCC CAG CC-3′	5′-CAT AAC TGT GTT CCC GAG GTG TC-3′
MCP-1	5′-AAG AGA ATC ACC AGC AGC AGG T-3′	5′-TTC TGG ACC CAT TCC TTA TTG G-3′
HO-1	5′-TCA GTC CCA AAC GTC GCG GT-3′	5′-GCT GTG CAG GTG TTG AGC C-3′
B1R	5′-CCA TAG CAG AAA TCT ACC TGG CTA AC-3′	5′-GCC AGT TGA AAC GGT TCC-3′
B2R	5′-ATG TTC AAC GTC ACC ACA CAA GTC-3′	5′TGG ATG GCA TTG AGC CAA C-3′
HPRT	5′-CTC ATG GAC TGA TTA TGG ACA GGA C-3′	5′-GCA GGT CAG CAA AGA ACT TAT AGC C-3′

Oligonucleotides sequence.

### Statistical Analysis

All data were described as mean±S.E.M. Different results among groups were compared using Kruskal-Wallis One Way Analysis of Variance on Ranks (ANOVA) or by the T-test. Results were considered significant when p<0.05. Survival curves were estimated by the Kaplan-Meier test. All statistical analyses were performed with the aid of SigmaStat Statistical Software 2.0 (Jandel Corporation, TX, USA).

## Results

### Bradykinin receptor expression in renal IRI

**Figure 1:**
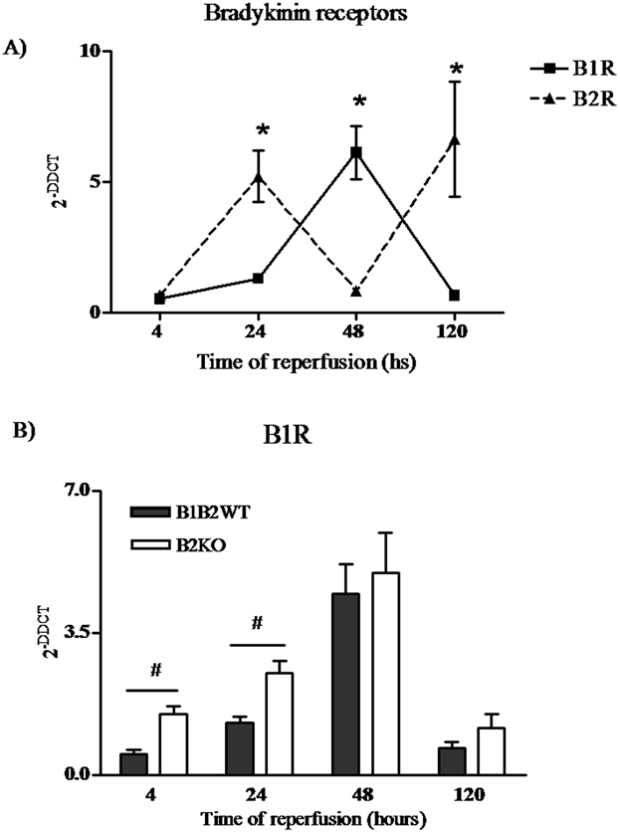
Renal IRI and bradykinin receptors expression. Bradykinin receptors were analyzed by real-time PCR. In B1B2WT, receptors expressions were cross-modulated (A). In B2KO, B1R expression was increased at 4 and 24 hours (B). Statistical analyses were performed by ANOVA. *B1R *versus* B2R, p<0.05. # B2KO *versus* B1B2WT, p<0.05.

B1KO, B2KO, B1B2KO and wild-type mice were subjected to renal IRI and kidney expression of bradykinin receptors were analyzed at 4, 24, 48 and 120 h of reperfusion. In wild-type mice, receptors expressions were alternated (Figure 1A), suggesting a cross-modulation between them. In B2KO, B1R expression was increased at 4 and 24 hours (Figure 1B). In B1KO mice, B2R expression was not modified (data not shown).

**Figure 2:**
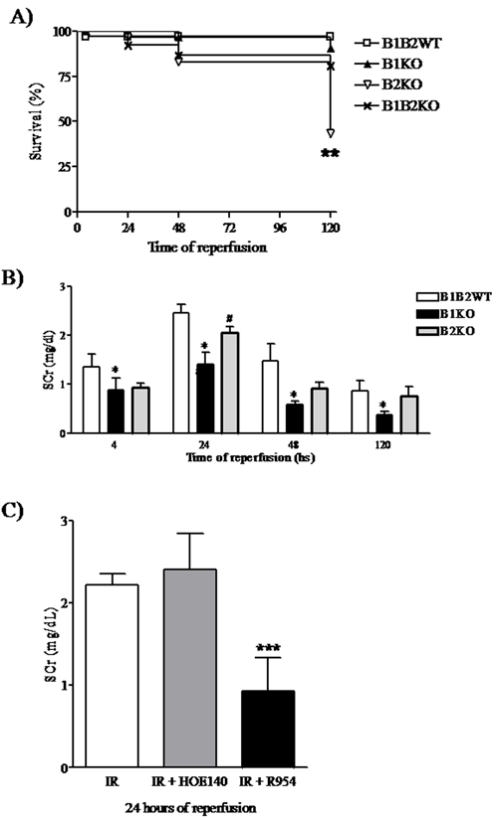
Animal survival and renal function after bradykinin receptor deletion or antagonism. Animal survival was assessed by Kaplan-Meier curve for 5 days after reperfusion. B2KO had the lowest survival at 120 hours of reperfusion (A). Renal function was estimated by serum creatinine levels measured by modified Jaffé method. B1KO protection was observed at all times, while B2KO protection was only seen at 4 and 24 hours (B). Thirty minutes prior to the ischemic insult, wild-type animals were treated with B1R or B2R antagonists (R-954 and HOE-140, respectively, both at 200 µg/kg) via i.p and plasmatic creatinine levels were assayed at 24 hours of reperfusion. Only the R-954 treatment resulted in lower SCr levels (C). Statistical analyses were performed using ANOVA. *B1KO *versus* B1B2WT, p<0.05 and #B2KO *versus* B1B2WT, p<0.05. ***IR+R-954 *versus* IR and IR+HOE-140, p<0.05.

Bradykinin receptor importance on survival was also analyzed and mortality of B2KO mice was increased at 120h post ischemia (Figure 2A).

### Modulation of renal function by Bradykinin receptors deletion or antagonism

All animals subjected to renal ischemia showed a significant rise in serum creatinine levels (sCr) after 4, 24 and 48 hours of reperfusion, in comparison to *sham*-operated (data not shown). All values returned to normal at 120 hours (Figure 2B).

B1KO had a significant reduction in sCr at all times analyzed, meanwhile B2KO mice presented lower sCr values only at 4 and 24 h. These results showed that B1R deletion was beneficial throughout different reperfusion times; meanwhile protection under B2R absence was only seen at the beginning of this process. Moreover, B1R antagonism (R-954) significantly reduced sCr levels at 24 hours (0.93±0.40 in treated *versus* 2.22±0.14 mg/dL in untreated animals, p = 0.005), which was not observed by B2R antagonism (HOE-140) (Figure 2C). Since a significant protection was only achieved by B1R antagonism, we decided to analyze whether its agonist (DABK) would have an impact on renal IRI. In this case, sCr levels were unaffected (2.22±0.14 in untreated *versus* 1.95±0.20 mg/dL in treated animals).

### Bradykinin receptors and apoptosis after renal IRI

**Figure 3:**
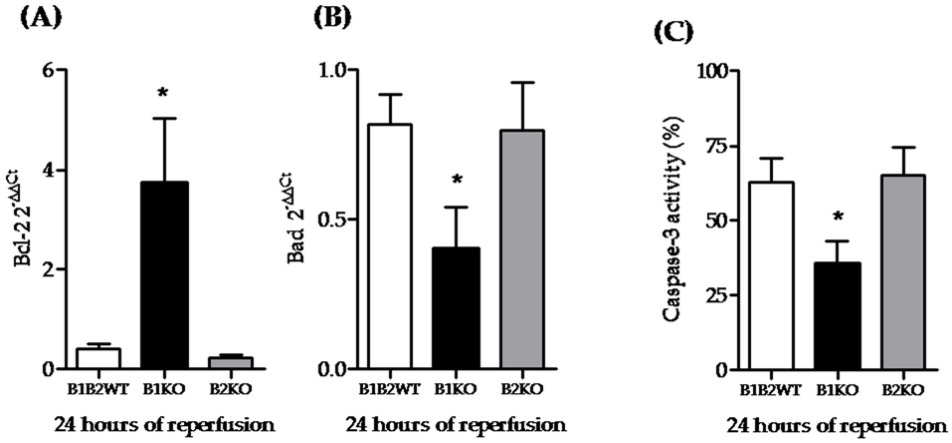
Cell death modulation under B1R-knockout. Apoptosis was estimated by Bcl-2 and Bad expression and caspase-3 activity, at 24 hours of reperfusion. Bcl-2 and Bad expression were measured by real-time PCR, and caspase-3 activity by fluorimetric assay. B1KO animals presented higher Bcl-2 expression (A) along with lower Bad expression (B) and caspase-3 activity (C), indicating a lower degree of apoptosis. Statistical analyses were performed using ANOVA.* B1KO *versus* B1B2WT and B2KO, p<0.05.

Cell death was estimated by the anti-apoptotic Bcl-2 and the pro-apoptotic Bad mRNA expression and caspase-3 activity, a central protease in apoptosis that is induced by renal ischemia. We observed that B1KO had increased Bcl-2 (Figure 3A) and decreased Bad (Figure B) expressions compared to wild-type and B2KO animals. Furthermore, caspase-3 activity of B1KO was significantly lower than wild type and B2KO (Figure 3C). These results thereby indicate that B1KO protection was associated with lower apoptosis.

### Pro and anti-inflammatory molecule expression in B1R knockout or antagonism after renal IRI

**Figure 4:**
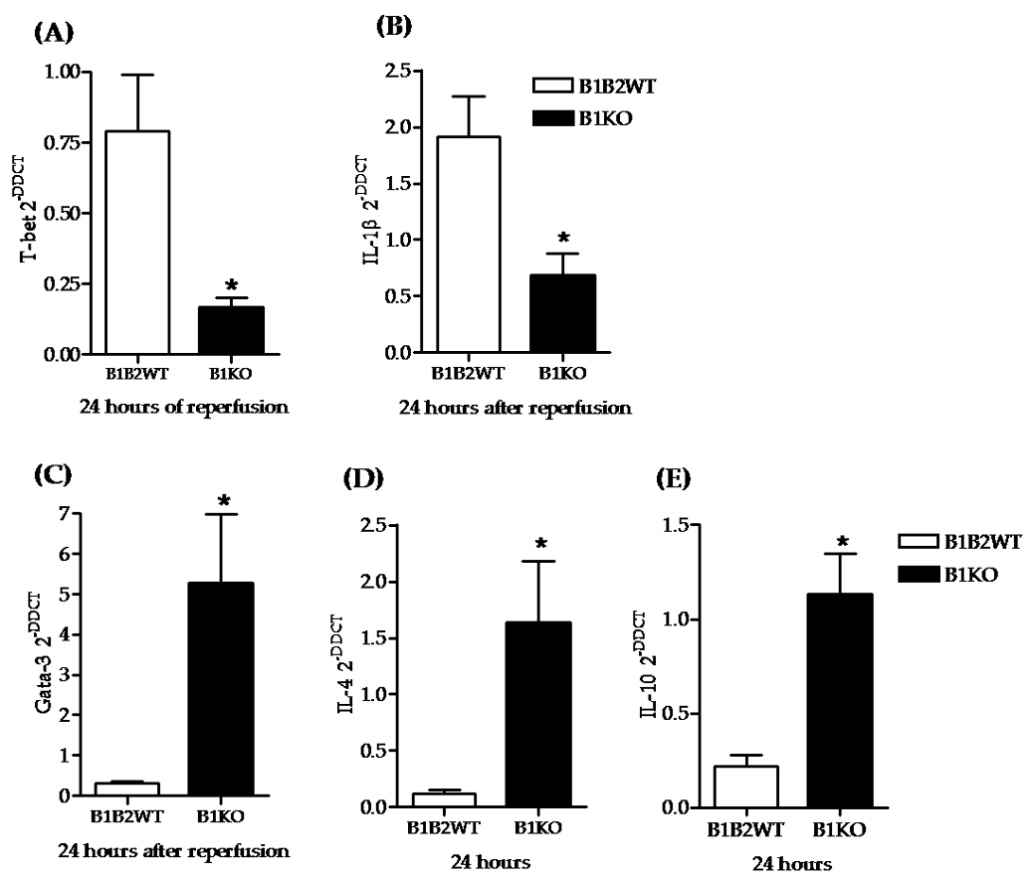
Pro and anti-inflammatory molecule expression in B1KO and wild type animals. All molecule expressions were measured by real-time PCR at 24 hours of reperfusion. B1KO group had lower pro-inflammatory molecule expression (T-bet and IL-1β) (A and B) and higher anti-inflammatory response (GATA-3, IL-4 and IL-10) (C, D and E). Statistical analyses were performed using the t-test.* B1KO *versus* B1B2WT, p<0.05.

**Figure 5:**
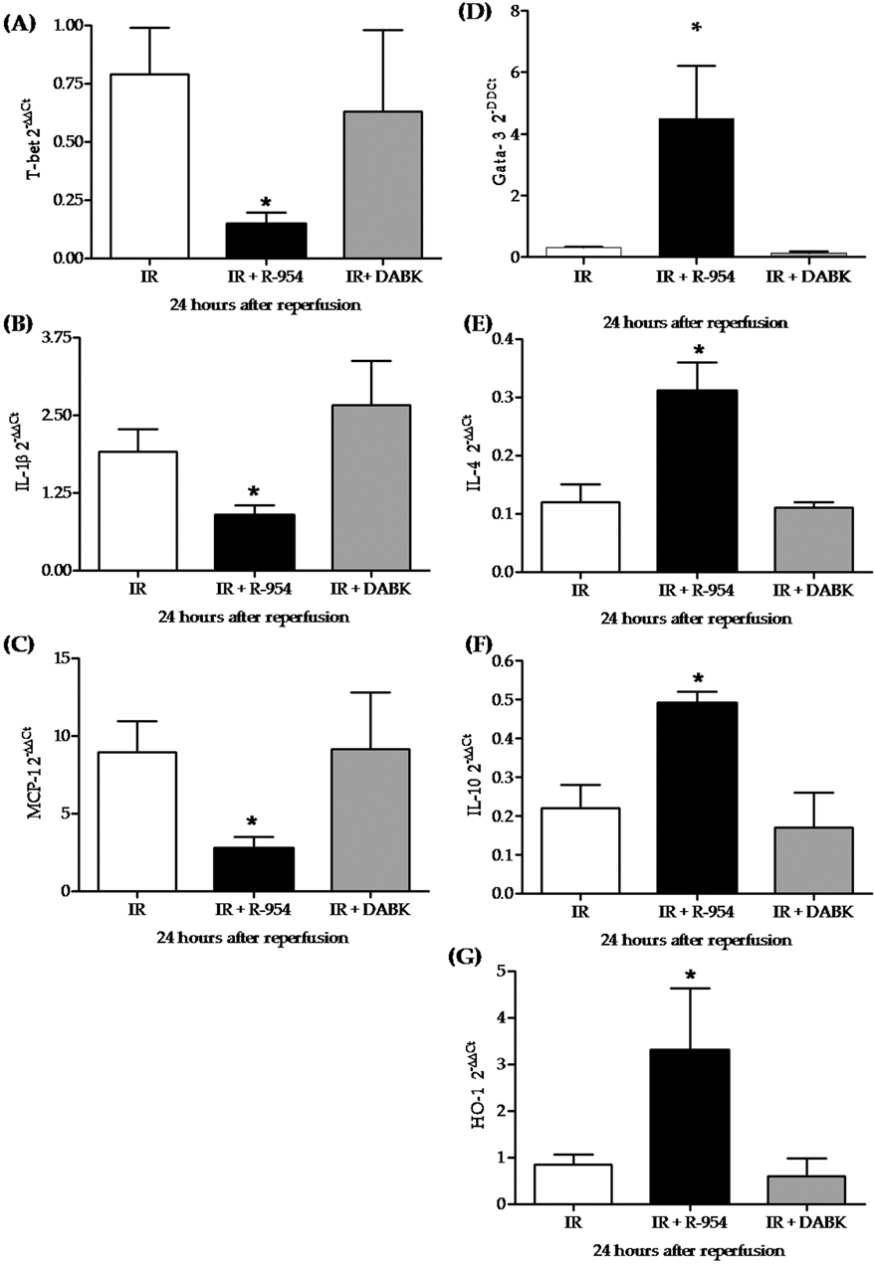
Pro and anti-inflammatory molecules expression after B1R antagonist and agonist treatment. All molecule expressions were estimated by real-time PCR at 24 hours of reperfusion. B1R antagonism (R-954) resulted in lower pro-inflammatory molecule expression (T-bet, IL-1β and MCP-1) (A, B and C) and higher anti-inflammatory response (GATA-3, IL-4, IL-10 and HO-1) (D, E, F and G). Molecule expression after B1R agonism (DABK) were similar to non-treated mice. Statistical analyses were performed using ANOVA.*IR+HOE-140 *versus* IR, p<0.05.

Pro and anti-inflammatory molecules were measured at 24 hours of reperfusion in B1R knockout or antagonist-treated mice. We observed that the pro-inflammatory transcriptional factor T-bet and the cytokines IL-1β were significantly reduced by B1R knockout (Figure 4a and b) and antagonism (Figure 5a and b). In the other hand, the anti-inflammatory components GATA-3, IL-4 and IL-10 were increased in knockout mice (Figure 4c, d and e) and R-954 treated group (Figure 5d, e and f). We previously have observed that B1R deletion increased HO-1 and decreased MCP-1 expression [27]. Herein, similar results were achieved by B1R antagonism (Figure 5c and g). B1R agonist was not to modify the expression of any molecule analyzed (Figure 5).

**Figure 6:**
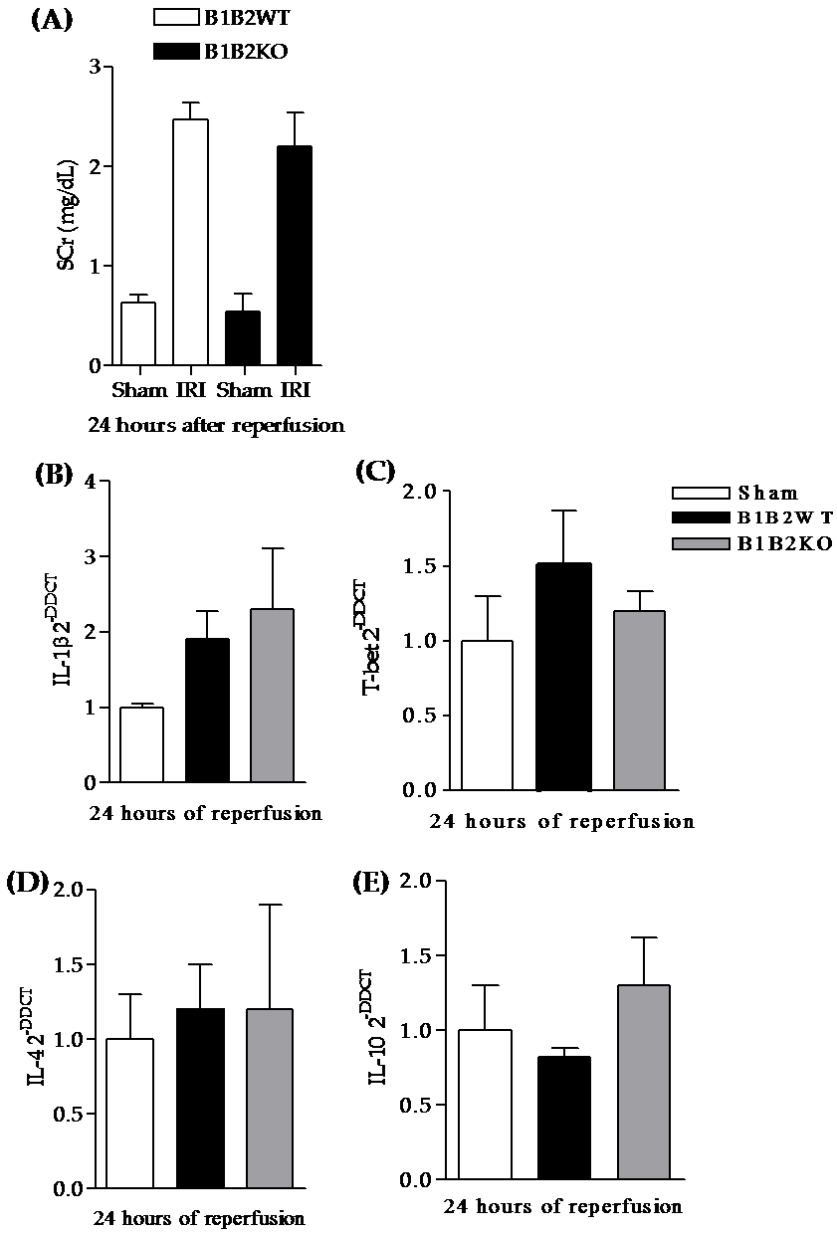
B1B2KO renal function and expression of pro and anti-inflammatory molecules. Renal function was estimated by serum creatinine levels measured using the modified Jaffé method. At 24 hours of reperfusion, B1B2KO presented high levels of serum creatinine and they were similar to wild-type mice (A). Thus, no renal protection was observed under simultaneous receptors deletion. Inflammatory molecule expressions were measured by real-time PCR. IL-1β (B), T-bet (C), IL-4 (D) and IL-10 (E) expressions were similar between the double-knockout strain and wild-type mice. Statistical analyses were performed using the T-test.* B1B2KO *versus* B1B2WT, p<0.05.

Renal function and the expression of some cytokines were also studied in B1B2KO mice. At 24 hours after IRI, these animals presented high levels of sCr (2.47±0.17 mg/dL in B1B2KO *versus* 2.22±0.14 mg/dL in B1B2WT) and thus, they were not protected from injury. They also presented a pro-inflammatory profile similar to wild-type animals (Figure 6b, c, d and e).

## Discussion

A previous study by our group demonstrated that bradykinin receptors may influence tissue outcome in renal IRI [27]. B2R are constitutively expressed, while B1R are inducible. In the present study, IRI induced B1R expression and a cross-modulation between receptors was observed. Cross-modulation has been previously described in different experimental models of intestinal [28], cardiac [29]–[31] and renal [31], [32] insults.

Our previous work has demonstrated that sCr levels, measured 24 hours after renal ischemia, were much lower in B1KO than in wild-type mice [27]. In this study, B1KO protection was confirmed at all stages. Moreover, we found that B1R antagonist R-954 also able to reduce sCr levels.

Concerning B2R and renal IRI, our previous paper showed that sCr levels were slightly lower in B2KO when compared to wild-type mice. However, this difference did not reach statistical significance. Here, the use of a larger number of B2KO animals resulted in a significant decreased of sCr exclusively at the beginning of reperfusion. In contrast, this protection was not mimicked by B2R antagonism and was not associated with any inflammatory molecule here analyzed. This led us to believe that B2KO protection might rely on expression and function of other molecules not measured here. In agreement with this, a previous study have shown that in renal IRI, early B2R deletion was associated with decreased serum creatine levels, ROS production, cell death and TNF-α and MCP-1 expression [23]. However, in the study of Chiang et al. [23] animals were subjected to a less severe IRI, which may explain the slight difference of renal dysfunction and tissue responses between our studies. Even more, they only observed tissue outcome at 24 hours of reperfusion, herein the analyses were made at 4, 24, 48 and 120 hours.

The 24-hour post ischemia period is commonly considered as the most deleterious in renal IRI, corresponding with the highest probability of cell death. Serum creatinine levels of all ischemic groups analyzed peaked at this 24 hr period, indicating that maximum renal damage occurred. We evaluated several molecules associated with cell death at 24 hrs and found their expression to be decreased in B1KO mice. Moreover, we found that the renal protection observed in B1KO was associated with a lower degree of apoptosis. Thus, B1R deletion may protect renal cells from death during IRI.

Concerning the immunological aspect of renal IRI, T lymphocytes are considered important mediators of this injury, since T cell depletion significantly improves renal function [4], [6], [7]. In this context, CD4 T cells have been a focus of research due to their differentiation to either a Th1 (pro inflammatory) or a counterbalancing Th2 (anti inflammatory) profile. In renal IRI, animals lacking the transcriptional factor of the Th2 profile (STAT-6) develop more severe renal damage, while deletion of the Th1 transcriptional factor STAT-4 results in mild protection. Furthermore, activation of the Th2 transcriptional factor GATA-3 followed by IL-4, IL-5, IL-6, IL-10 and IL-13 production was shown to be protective, whereas higher expression of the transcriptional factor T-bet, and IL-12, IL-1β and IFN-γ was deleterious [6], [8], [33]–[35]. All of these studies point out that tissue outcome after renal IRI may be influenced by anti and pro inflammatory responses.

To investigate possible immunological responses that were involved in B1KO protection, the expression of some anti and pro inflammatory components were analyzed in the kidneys. We found that B1R gene deletion or antagonism resulted in higher expression of the anti-inflammatory GATA-3, IL-4 and IL-10 and lower transcription of the pro-inflammatory T-bet and IL-1β. Other studies have associated B1R activation with enhanced inflammation [29], [36], [37]. Ni and colleagues demonstrated in a rat model that overexpression of B1 receptors exacerbated paw edema induced by carrageenan, and rendered transgenic mice more susceptible to LPS-induced endotoxic shock [28]. Liesmaa and collaborators identified increased expression of B1R in endothelium of failing human hearts. They demonstrated that B1R knockout mice, in contrast to B2R knockout mice, did not spontaneously develop heart failure and presented an altered inflammatory response, suggesting that B1R play an essential role in the initiation of inflammation following myocardial ischemia [37]. Engagement of the B1R has pro-inflammatory effects, including promotion of leukocyte traffic, edema and pain. Moreover, it has been demonstrated that B1R activation can enhance the release of prostaglandins [16], mast cell mediators [15], cytokines, mainly IL-1β [18], [19] and chemokines and leukotrienes in different cell types [20]–[22].

Our results suggest a major influence of B1R in renal ischemia, since treatment with its antagonist resulted in functional improvement. B1R antagonist also changed the profile of pro and anti inflammatory molecules, whose expression was induced by IRI. Animals treated with R-954 showed an increased anti-inflammatory cytokine profile, whereby GATA-3, IL-4 and IL-10 were up regulated while T-bet and IL-1β expressions were down regulated.

Even more, the expression of MCP-1 was reduced while HO-1 was up regulated by B1R antagonism, confirming previous observations from our group that showed a similar expression profile in B1KO mice [27]. Ischemic organ outcome is determined mainly by the tuned balance between aggression and cytoprotection. HO-1 is a protective enzyme able to generate carbon monoxide (CO), biliverdin and free iron, through heme metabolism (reviewed in Camara NO and Soares MP, 2005 [38]), which can be protective against ischemic insults [39]. Sacerdoti and colleagues have recently demonstrated that HO-1 expression is inversely correlated with MCP-1 [40].

Herein, we also showed that simultaneous deletion of both receptors resulted in extremely high levels of sCr and in a pro-inflammatory profile. In concordance with this, worse prognoses of double knockout mice were also observed in pancreas [32] and in another study of renal [24] IRI. Kakoki et al. linked this deleterious effect to an enhanced pattern of oxidative stress [24]. Here, we demonstrated that B1B2KO mice expressed a pro-inflammatory phenotype that might explain part of their renal dysfunction.

Taken together, our results showed that the protection found under B1R gene deletion or antagonism involves a shift towards an anti-inflammatory profile, concomitant with down regulation of pro-inflammatory molecules and upregulation of anti-inflammatory ones. Since B1R antagonism proved to be protective, we believe that this may represent a new therapeutic strategy against renal IRI.
