# Correction: Bradykinin B1 Receptor Antagonism Is Beneficial in Renal Ischemia-Reperfusion Injury

**DOI:** 10.1371/annotation/bde714c3-7f6d-4f03-83b3-e291005c3a39

**Published:** 2008-09-05

**Authors:** Pamella H. M. Wang, Gabriela Campanholle, Marcos A. Cenedeze, Carla Q. Feitoza, Giselle M. Gonçalves, Richardt G. Landgraf, Sonia Jancar, João B. Pesquero, Alvaro Pacheco-Silva, Niels O. S. Câmara

The first word of the title is misspelled. This also affect the article's citation. The correct title should be: Bradykinin B1 Receptor Antagonism Is Beneficial in Renal Ischemia-Reperfusion Injury. The corrected citation is: Wang PHM, Campanholle G, Cenedeze MA, Feitoza CQ, Gonçalves GM, et al. (2008) Bradykinin B1 Receptor Antagonism Is Beneficial in Renal Ischemia-Reperfusion Injury. PLoS ONE 3(8): e3050. doi:10.1371/journal.pone.0003050.

